# Lumbar Puncture in Thrombocytopenia: The Floor Is Not Firm

**DOI:** 10.7759/cureus.42019

**Published:** 2023-07-17

**Authors:** Michael Kozak, David R Hallan, Mason Stoltzfus, Elias Rizk

**Affiliations:** 1 Neurosurgery, Penn State College of Medicine, Hershey, USA

**Keywords:** epidural blood patch, spinal hematoma, bleeding risk, thrombocytopenia, lumbar puncture (lp)

## Abstract

Objective

Lumbar puncture (LP) is a diagnostic procedure that accesses the spinal subarachnoid space to measure the opening pressure of the cerebrospinal fluid (CSF) and obtain samples of CSF for analysis. Although commonly performed, LPs are associated with the risk of morbidity and mortality. In addition, thrombocytopenia is thought to increase the risk of LP complications, particularly spinal bleeds. This study compares rates of complications among patients who received LPs with and without thrombocytopenia in hopes of establishing more evidence-based platelet thresholds for an LP.

Methods

The TriNetX multi-institutional electronic health record database was used to perform a retrospective propensity score-matched analysis of clinical outcomes of two cohorts of patients who underwent LPs - those with thrombocytopenia (defined as a platelet level of 10,000-50,000 platelets {plts}/μL) and those without thrombocytopenia. The outcomes of interest were the new occurrence of subdural hematoma, epidural hematoma, subarachnoid hemorrhage, receipt of a blood patch, new onset of paralysis, and requirement of spinal decompression.

Results

The risk of developing a spinal bleed following an LP was 1.496% (42 of 2,808) for the cohort with thrombocytopenia versus 1.09% (31 of 2,843) for the cohort without thrombocytopenia. The risk difference, risk ratio, and odds ratio of patients from these two cohorts experiencing a spinal bleed following an LP were insignificant at 0.05. The risk of receiving a blood patch following an LP was 7.844% for those with thrombocytopenia compared to 1.421% for those without thrombocytopenia. The odds ratio of receiving a blood patch between the two cohorts was 5.906, significant to the 0.05 level (95% CI: 4.213-8.279). There was no significant difference in the cohorts’ risk of developing paralysis or requiring spinal decompression following an LP.

Conclusion

In support of recent findings against conventional platelet count thresholds prior to LP, it was observed in the present study that the incidence of post-LP spinal bleeding in the 30 days after LP is not associated with platelet counts below the guideline threshold of 50,000 plts/μL. Patients with thrombocytopenia are also not significantly more likely to require spinal decompression or develop new onset paralysis. However, thrombocytopenia is associated with a significantly increased likelihood of receiving a blood patch following an LP.

## Introduction

Lumbar puncture is a common diagnostic procedure used across medical disciplines to analyze cerebrospinal fluid and its opening pressure. It also serves as a therapeutic procedure for pathologically increased intracranial pressure. European analyses have shown that more than 1% of all hospital contacts involve a lumbar puncture [1]. An LP is most commonly used as a diagnostic procedure in the work-up of suspected meningitis [2]. LP-mediated CSF analysis facilitates rapid determination of the causative classes of pathogens for meningitis [3], with a sensitivity and specificity of 98% and 75%, respectively, for bacterial meningitis [4].

While meningitis affects all populations including infants, the elderly, and immunosuppression patients are at higher risk of infection. LPs are most frequently performed on immunosuppressed patients, many of whom suffer from hematologic malignancies with concomitant thrombocytopenia.

The most frequently reported complications of an LP are backache (25%), post-LP headache (22%), and radicular pain (5%). These symptoms are often caused by infection, bleeding, cerebral herniation, late onset of epidermoid tumors of the thecal sac, and nerve injury [5]. Bleeding in the subarachnoid space is associated with significant morbidity relative to other LP-associated complications and warrants significant pre-procedure consideration.

Thrombocytopenia and elevated intracranial pressure (ICP) almost always necessitate a work-up prior to lumbar puncture [6]. Approximately 10% of bedside LPs are considered "traumatic" and rupture blood vessels that can bleed into the subarachnoid space [7,8]. Due to this, some guidelines recommend a platelet value above 50,000 platelets (plts)/μL before proceeding with a lumbar puncture [6,7,9,10].

Recently, evidence has been mounting against a platelet threshold prior to an LP; until recently, the data was mostly from single-center studies of populations with hematological malignancies that indicated no increased risk for post-LP complications [9]. However, case series of neuraxial anesthesia during parturition and for patients with hematological malignancies undergoing LPs from the 1980s through the 2010s provide consistent evidence for a re-examination of platelet guidelines [7,9,11,12].

This study aimed to leverage a large, multi-institutional healthcare database of patient information to determine the relative safety of an LP by comparing outcomes of lumbar punctures performed on patients with and without thrombocytopenia.

## Materials and methods

This is a retrospective case-control study using a multi-institutional healthcare database, TriNetX, to collect data from up to 68 different global healthcare organizations on patients who received lumbar punctures with and without thrombocytopenia. The TriNetX United States (US) Collaborative Network, containing information from 86,401,400 de-identified patients collected over 20 years, was accessed on September 26, 2022, to stratify patients into two cohorts. Cohort 1 included patients who had received an LP, had a diagnosis of thrombocytopenia with a platelet level of 10,000 to 50,000 plts/μL, and had not received antiplatelet medications, anticoagulant medications, or platelet transfusions in the month prior to an LP. Cohort 2 included patients without a diagnosis of thrombocytopenia and without receipt of antiplatelet or anticoagulant medications in the month prior to an LP. The patients in cohort 2 also had propensity scores matched 1:1 to those in cohort 1, using common baseline demographics and comorbidities, such as age, gender, race, cirrhosis, hypertension, diabetes mellitus, obesity, nicotine use, alcohol abuse, chronic lower respiratory disease, atrial fibrillation, atrial flutter, heart failure, ischemic heart disease, peripheral vascular disease, thrombocytopenia, immune thrombocytopenic purpura, qualitative platelet defects, von Willebrand disease, disseminated intravascular coagulopathy, hereditary factor XI deficiency, hereditary factor VIII deficiency (hemophilia A), hereditary factor IX deficiency (hemophilia B), activated protein C resistance, prothrombin gene mutation, antiphospholipid syndrome, lupus anticoagulant syndrome, using patient demographic information, diagnoses, and medications. The index date was set as the date of an LP. The TriNetX database is federated, thus an institutional review board approval for this study has been waived.

Multiple outcomes were compared across these two cohorts, including the occurrence of epidural hematoma, subdural hematoma, subarachnoid hemorrhage, hemiparesis, receiving a blood patch, and undergoing spinal decompression.

All statistical analysis was performed using the TriNetX online platform. Descriptive measures, such as means with standard deviations and proportions, were used to describe patient characteristics. For each outcome, the risk ratio, relative risk, and odds ratio were calculated to estimate the effects of thrombocytopenia on the outcomes. A priori-defined two-sided alpha value of <0.05 was used for statistical significance.

## Results

Among all qualifying patients in the database, there were 2,920 patients (cohort 1) with thrombocytopenia below the recommended threshold (platelets: 10,000-50,000 plts/μL) without receipt of a platelet transfusion, antiplatelet medication, or anticoagulant medication within the past month who underwent an LP. In addition, 221,592 patients in the database underwent an LP and did not receive a platelet transfusion, antiplatelet medication, or anticoagulant medication within one month of the procedure (cohort 2). Mean age, gender ratio, ethnicity, and comorbid conditions were substantially dissimilar in both groups prior to propensity matching (Table 1).

**Table 1:** Patient demographics prior to propensity matching. The table shows the baseline demographics for patients who underwent an LP with a diagnosis of thrombocytopenia and without receipt of antiplatelet medications, anticoagulant medications, or platelet transfusions in the month prior to an LP (cohort 1; N=2,920) and for those without thrombocytopenia and no receipt of antiplatelet or anticoagulant medications in the month prior to an LP (cohort 2; N=221,592), before propensity score matching. LP: lumbar puncture

Cohort	Code	Characteristic	Mean±SD	Patients	Percentage of cohort	p-Value	Std. diff.
1	AI	Age at index	40.2±25.1	2,920	100%	<0.001	0.217
2			34.9±24.1	221,592	100%		
1	2,106-3	White	-	1,839	62.98%	0.164	0.026
2				142,309	64.22%		
1	2,054-5	Black or African American	-	405	13.87%	<0.001	0.110
2				39,598	17.87%		
1	2,028-9	Asian	-	46	1.58%	0.519	0.013
2				3,845	1.74%		
1	M	Male	-	1,642	56.23%	<0.001	0.258
2				96,305	43.46%		

Baseline comorbid conditions for which cardiovascular and hematologic function may be altered are presented in Table 2, from which it can be seen that nearly all of these conditions were substantially dissimilar prior to propensity matching. After a propensity-matched data analysis was performed, 2,909 patients remained in both cohorts (Table 3).

**Table 2:** Selected baseline comorbidities prior to propensity matching. The table shows the selected baseline comorbidities for patients who underwent an LP with a diagnosis of thrombocytopenia and without receipt of antiplatelet medications, anticoagulant medications, or platelet transfusions in the month prior to an LP (cohort 1; N=2,920) and for those without thrombocytopenia and no receipt of antiplatelet or anticoagulant medications in the month prior to an LP (cohort 2; N=221,592), before propensity score matching. LP: lumbar puncture

Cohort	Code	Characteristic	Patients	Percentage of cohort	p-Value	Std. diff.
1	I10-I16	Hypertensive diseases	967	33.12%	<0.001	0.239
2			49,840	22.49%		
1	D69.6	Thrombocytopenia, unspecified	1,231	42.16%	<0.001	1.057
2			6,784	3.06%		
1	E08-E13	Diabetes mellitus	479	16.40%	<0.001	0.203
2			21,272	9.60%		
1	F17	Nicotine dependence	415	14.21%	<0.001	0.108
2			23,609	10.65%		
1	D65	Disseminated intravascular coagulation (defibrination syndrome)	181	6.20%	<0.001	0.348
2			385	0.17%		
1	D68.62	Lupus anticoagulant syndrome	20	0.69%	<0.001	0.060
2			596	0.27%		
1	J40-J47	Chronic lower respiratory diseases	410	14.04%	0.029	0.040
2			28,108	12.69%		
1	I20-I25	Ischemic heart diseases	345	11.82%	<0.001	0.230
2			11,991	5.41%		
1	E65-E68	Overweight, obesity and other hyperalimentation	339	11.61%	0.159	0.026
2			23,922	10.80%		
1	D69.3	Immune thrombocytopenic purpura	61	2.09%	<0.001	0.185
2			333	0.15%		
1	D68.61	Antiphospholipid syndrome	23	0.79%	<0.001	0.068
2			640	0.29%		
1	I48	Atrial fibrillation and flutter	240	8.22%	<0.001	0.265
2			5,195	2.34%		
1	F10.1	Alcohol abuse	179	6.13%	<0.001	0.188
2			5,231	2.36%		
1	F10.2	Alcohol dependence	174	5.96%	<0.001	0.210
2			4,210	1.90%		
1	I50	Heart failure	295	10.10%	<0.001	0.289
2			6,687	3.02%		
1	I73	Other peripheral vascular diseases	90	3.08%	<0.001	0.072
2			4,320	1.95%		
1	D69.1	Qualitative platelet defects	20	0.69%	<0.001	0.100
2			190	0.09%		
1	D68.51	Activated protein C resistance	19	0.65%	<0.001	0.053
2			639	0.29%		
1	K74	Fibrosis and cirrhosis of the liver	267	9.14%	<0.001	0.380
2			2,152	0.97%		
1	D68.52	Prothrombin gene mutation	16	0.55%	0.002	0.047
2			555	0.25%		
1	D68.0	Von Willebrand disease	10	0.34%	<0.001	0.060
2			154	0.07%		
1	D66	Hereditary factor VIII deficiency	10	0.34%	<0.001	0.070
2			86	0.04%		
1	D67	Hereditary factor IX deficiency	10	0.34%	<0.001	0.079
2			24	0.01%		
1	D68.1	Hereditary factor XI deficiency	0	0%	<0.001	0.079
2			20	0.01%		

**Table 3:** Demographic data after propensity matching. Demographics for patients who underwent an LP with a diagnosis of thrombocytopenia and without receipt of antiplatelet medications, anticoagulant medications, or platelet transfusions in the month prior to an LP (cohort 1; N=2,909) and those without thrombocytopenia and no receipt of antiplatelet or anticoagulant medications in the month prior to an LP (cohort 2; N=2,909), after propensity score matching. LP: lumbar puncture

Cohort	Code	Characteristic	Mean±SD	Patients	Percentage of cohort	p-Value	Std. diff.
1	AI	Age at index	40.2±25.1	2,909	100%	0.451	0.020
2			40.7±25.5	2,909	100%		
1	2,106-3	White	-	1,831	62.94%	0.386	0.023
2				1,799	61.84%		
1	2,054-5	Black or African American	-	403	13.85%	0.466	0.019
2				384	13.20%		
1	2,028-9	Asian	-	46	1.58%	0.379	0.023
2				38	1.31%		
1	M	Male	-	1,633	56.14%	0.153	0.038
2				1,687	57.99%		

Propensity matching generated not dissimilar cohorts across the selected comorbidities considered to be most consequential to hematologic and thrombotic outcomes excluding a diagnosis of thrombocytopenia as a propensity control (Table 4). The clinical data are presented in Table 5, the clinical outcome time period for all outcomes is one month following LP.

**Table 4:** Comorbid patient characteristics after propensity score matching. The table shows comorbid conditions among patients who underwent an LP with a diagnosis of thrombocytopenia and without receipt of antiplatelet medications, anticoagulant medications, or platelet transfusions in the month prior to an LP (cohort 1; N=2,909) and those without thrombocytopenia and no receipt of antiplatelet or anticoagulant medications in the month prior to an LP (cohort 2; N=2,909), after propensity score matching. LP: lumbar puncture

Cohort	Code	Characteristic	Patients	Percentage of cohort	p-Value	Std. diff.
1	I10-I16	Hypertensive diseases	958	32.93%	0.128	0.040
2			1,013	34.82%		
1	D69.6	Thrombocytopenia, unspecified	1,221	41.97%	<0.001	0.808
2			270	9.28%		
1	E08-E13	Diabetes mellitus	475	16.33%	0.095	0.044
2			523	17.98%		
1	F17	Nicotine dependence	410	14.09%	0.595	0.014
2			396	13.61%		
1	D65	Disseminated intravascular coagulation (defibrination syndrome)	171	5.88%	0.062	0.049
2			139	4.78%		
1	D68.62	Lupus anticoagulant syndrome	20	0.69%	0.621	0.013
2			17	0.58%		
1	J40-J47	Chronic lower respiratory diseases	407	13.99%	0.297	0.027
2			435	14.95%		
1	I20-I25	Ischemic heart diseases	341	11.72%	0.468	0.019
2			359	12.34%		
1	E65-E68	Overweight, obesity and other hyperalimentation	336	11.55%	0.534	0.016
2			321	11.04%		
1	D69.3	Immune thrombocytopenic purpura	58	1.99%	0.703	0.010
2			54	1.86%		
1	D68.61	Antiphospholipid syndrome	23	0.79%	0.341	0.025
2			17	0.58%		
1	I48	Atrial fibrillation and flutter	236	8.11%	0.668	0.011
2			245	8.42%		
1	F10.1	Alcohol abuse	175	6.02%	0.869	0.004
2			178	6.12%		
1	F10.2	Alcohol dependence	172	5.91%	0.547	0.016
2			183	6.29%		
1	I50	Heart failure	289	9.94%	0.089	0.045
2			329	11.31%		
1	I73	Other peripheral vascular diseases	90	3.09%	0.270	0.029
2			76	2.61%		
1	D69.1	Qualitative platelet defects	19	0.65%	0.491	0.018
2			15	0.52%		
1	D68.51	Activated protein C resistance	19	0.65%	0.872	0.004
2			20	0.69%		
1	K74	Fibrosis and cirrhosis of the liver	261	8.97%	0.715	0.010
2			269	9.25%		
1	D68.52	Prothrombin gene mutation	16	0.55%	0.857	0.005
2			15	0.52%		
1	D68.0	Von Willebrand disease	10	0.34%	>0.999	<0.001
2			10	0.34%		
1	D66	Hereditary factor VIII deficiency	10	0.34%	>0.999	<0.001
2			10	0.34%		
1	D67	Hereditary factor IX deficiency	10	0.34%	>0.999	<0.001
2			10	0.34%		
1	D68.1	Hereditary factor XI deficiency	0	0%	-	-
2			0	0%		

**Table 5:** Clinical outcome comparison between those who underwent an LP with thrombocytopenia (cohort 1) or without thrombocytopenia (cohort 2). The table shows the measures of association for patients who underwent an LP with thrombocytopenia (cohort 1) or without thrombocytopenia (cohort 2) concerning clinical outcomes (excluding all patients with outcomes prior to the time window).

Cohort			Patients in cohort	Patients with outcome	Percentage (%)
Outcome: occurrence of subdural hematoma, epidural hematoma, and subarachnoid hemorrhage					
1	LP and thrombocytopenia		2,808	42	1.496
2	LP and no thrombocytopenia		2,843	31	1.09
Odds ratio		1.377	95% CI: 0.863, 2.197)	p-Value: 0.1791	
Outcome: receipt of blood patch					
1	LP and thrombocytopenia		2,792	219	7.844
2	LP and no thrombocytopenia		2,886	41	1.421
Odds ratio		5.906	95% CI: 4.213, 8.279	p-Value: <0.0001	
Outcome: occurrence of new paralysis					
1	LP and thrombocytopenia		2,751	63	2.29
2	LP and no thrombocytopenia		2,697	74	2.744
Odds ratio		0.831	95% CI: 0.591, 1.167	p-Value: 0.2855	
Outcome: underwent spinal decompression					
1	LP and thrombocytopenia		2,890	10	0.346
2	LP and no thrombocytopenia		2,889	10	0.346
Odds ratio		1	95% CI: 0.415, 2.405	p-Value: 0.9994	

The percentage of patients that experienced a spinal bleed, including subdural hemorrhage, subarachnoid hemorrhage, and epidural hemorrhage, in the month after LP was similar between the cohorts with 42 of 2,808 (1.496%) patients in the thrombocytopenic cohort and 31 of 2,843 (1.09%) patients in the non-thrombocytopenic cohort experiencing spinal bleeds. The odds ratio of the cohorts experiencing the outcome was 1.377 (95% CI: 0.863-2.197) which was insignificant at 0.05.

The percentage of patients receiving a blood patch in the month following an LP was 7.844% in cohort 1 (thrombocytopenic) compared with 1.421% in cohort 2. The odds ratio of 5.906 was statistically significant at the 0.05 level (95% CI: 4.213-8.279).

The percentage of patients who experienced paralysis in the month after LP was also similar in both cohorts, with 63 of 2,751 (2.29%) patients in the thrombocytopenic cohort and 74 of 2,697 (2.744%) patients in the non-thrombocytopenic cohort experiencing paralysis. The odds ratio of the cohorts experiencing the outcome was 0.831 (95% CI: 0.591-1.167) which was insignificant at 0.05.

The percentage of patients who experienced spinal decompression in the month following an LP was 0.346% in both cohorts. The odds ratio of the cohorts for this outcome was one (95% CI: 0.415, 2.405) was insignificant at 0.05.

## Discussion

Most of the current literature relating platelet counts and the risk of complication following an LP focus on specific subpopulations of patients, such as adult and pediatric leukemia patients [9], patients with coagulopathies [2], and patients on long-term anticoagulants [10]. These studies and case reports helped set the standard platelet thresholds for all patients prior to an LP. 

If a patient does not meet the platelet threshold required for an LP, they may receive a platelet transfusion. However, platelet transfusions are associated with a degree of morbidity and mortality, the most common adverse effects being febrile (1/14) and hypersensitivity reactions (1/50) [7]. Therefore, evaluating the necessity and utility of meeting platelet parameters before performing LPs is pertinent.

The evidence for prophylactic transfusions is based on case reports of spinal hemorrhage following a lumbar puncture. Most occurred at platelet counts below 40,000 plts/μL. Still, spinal hemorrhage has been described at higher platelet counts, and the risk is higher in the setting of coagulopathy, traumatic taps, rapidly falling platelet counts, and hyperleukocytosis [13].

The American College of Physicians' (ACP) most recent 2015 guidelines note that LP bleeding complications are rare. However, bearing in mind that spinal bleeds are associated with potentially devastating neurologic sequelae, the ACP reasons that, in the absence of more robust published data supporting the safety of a lower threshold in adult patients, a fairly liberal platelet count threshold of 50,000 plts/μL is prudent. The 50,000 plts/μL threshold is only recommended for diagnostic or therapeutic LPs. Despite a lack of supportive data, a greater platelet count is often recommended for other procedures, such as epidural anesthesia. The ACP has graded this a "very weak recommendation" with "very low-quality evidence" [7]. Currently, evidence is mounting against requiring a platelet threshold prior to an LP; until recently, the data was mostly from single-center studies of populations with hematological malignancies that indicated no increased risk for post-LP complications [9].

In a recent, population-based cohort study of patients in the Danish National Registry with coagulopathies, there was not any statistically significant increased risk of spinal hematoma in 30 days after an LP among the coagulopaths (coagulopathies were defined as “platelet count lower than 150,000 plts/μL, international normalized ratio {INR} higher than 1.4, activated partial thromboplastin time {aPTT} longer than 39 seconds, or a combination of these parameters compared to matched peers without coagulopathies”). The overall risk of spinal hematoma in patients without coagulopathy was 0.2% versus 0.23% in patients with coagulopathy. This difference was not statistically significant, and the risk of spinal hematoma did not increase with the severity of coagulopathy. Furthermore, the incidence of spinal hematoma did not appear to increase based on the severity of the thrombocytopenia. The incidence of spinal hematoma was 0.2% for patients with a platelet count greater than 150,000 plts/μL, compared to 0.19% for patients with platelet counts between 51,000 and 100,000 plts/μL, 0.13% in patients with platelet counts between 31,000 and 50,000 plts/μL, 0.23% in patients with platelet counts between 11,000 and 30,000 plts/μL (two in 886), and 0% in patients with platelet counts between 1 and 10,000 plts/μL (zero in 221) [2].

An American, single-institution, retrospective cohort study using patient data from 2004 to 2018 compared patients receiving common antiplatelet agents up until receiving an LP and those who had discontinued all antiplatelet agents a month prior and found no significantly increased risk of hemorrhage or hematoma for those taking antiplatelet agents until the time of procedure compared with those who had discontinued antiplatelet agents four weeks before LP [14].

The Society for Interventional Radiology has incorporated these recent findings into its most recent guidelines, changing lumbar puncture from a moderate to a low-risk procedure in 2019 and recommending a new platelet threshold of at least 20,000 plts/μL required for LP [10]. The present study adds to the recent literature by exploring the association of thrombocytopenia with spinal bleeding and other post-LP morbidities for a critical reappraisal of the necessity of platelet threshold guidelines [10].

In agreement with the findings from the Danish National Registry, the present study did not identify an association between thrombocytopenia and an increased risk of subdural, epidural, or subarachnoid bleeding following an LP compared to patients without thrombocytopenia. The current study expands on the Danish study, demonstrating that an LP was not associated with an increased risk of spinal bleeding.

Additionally, in concordance with the American retrospective cohort study, the present study found no increased risk of spinal hematoma after LP in the patients with platelet counts below 50,000 plts/μL. This 50,000 plts/μL is well below the standard diagnostic value for thrombocytopenia of 150,000 plts/μL and below most current society guideline minimums for LP [13].

This study is not without limitations. The major limitation of this study is that it is retrospective in nature. The indication for an LP was not known. Due to the nature of the database, we do not have patient-level data on specific outcomes. We do not have access to imaging studies nor know the size of the spinal hemorrhage or the degree of spinal stenosis. The spinal hematoma could have caused the indications for spinal decompression. The exact time from an LP to hemorrhage is unknown. Lastly, some misidentification is inevitable in database studies.

## Conclusions

This study sought to look at the incidence of spinal bleeds after an LP in thrombocytopenic patients with a platelet count of 10,000-50,000 plts/μL versus patients with platelet counts above the commonly recommended threshold of 50,000 plts/μL at the time of LP. It was observed that no statistically significant association between low platelet counts and post-LP spinal bleeding at 30 days (one month) existed. Likewise, those with thrombocytopenia were no more likely to develop paralysis, nor to undergo spinal decompression, in the 30 days after an LP. Interestingly, low platelet count at the time of an LP was associated with a significantly increased likelihood of subsequently receiving a blood patch.
